# Analysis of emergency department visits for all reasons by adults with depression in the United States

**DOI:** 10.1186/s12873-020-00347-6

**Published:** 2020-06-22

**Authors:** Tyler Hill, Yun Jiang, Christopher R. Friese, Lynae A. Darbes, Christopher K. Blazes, Xingyu Zhang

**Affiliations:** 1 Department of Psychology, University of Michigan College of Literature, Science, and the Arts, Ann Arbor, MI USA; 2 Department of Systems, Populations, and Leadership, University of Michigan School of Nursing, Room 1177, 400 North Ingalls Building, Ann Arbor, MI 48109-5482 USA; 3 Department of Health Behavior and Biological Sciences, University of Michigan School of Nursing, Ann Arbor, MI USA; 4 Department of Emergency Medicine, University of Michigan School of Medicine, Ann Arbor, MI USA; 5 Department of Psychiatry, University of Michigan School of Medicine, Ann Arbor, MI USA

**Keywords:** Depression, Emergency medicine, National characteristics, Resource utilization

## Abstract

**Background:**

We aimed to characterize Emergency Department (ED) utilization and outcomes of patients with depression seeking emergency care for all reasons.

**Methods:**

Using 2014–2016 ED data from the National Hospital Ambulatory Medical Care Survey, we investigated demographics, ED resource utilization, clinical characteristics, and disposition of patients with depression versus those without depression.

**Results:**

Approximately 10,626,184 (11.4%) out of 92,899,685 annual ED visits were by patients with depression. ED patients with depression were mostly non-Hispanic White (74.0%) and were less likely to be male than patients without depression (aOR: 0.62; [95%] CI: 0.57–0.68). ED patients with depression were more likely to be admitted to the hospital (aOR: 1.50; CI: 1.38–1.63) than patients without depression. Among ED patients with depression, males were more likely than females to be seeking emergency care for psychiatric reasons (OR: 2.45; 95% CI: 2.10–2.87)) and to present with overdose/poisoning (OR: 1.46; CI: 1.03–2.05).

**Conclusions:**

We described the unique demographic, socioeconomic, and clinical characteristics of ED patients with depression, using the most comprehensive, nationally representative study to date. We revealed notable gender disparities in rates and reasons for admissions. The higher hospital and ICU admission rates of ED patients with depression suggests this population requires a higher level of emergency care, for reasons that remain poorly understood.

## Background

Depression represents an increasingly costly public health concern in the United States [1, 2]. An estimated 17.3 million adults (7.1%) in the US had at least one major depressive episode in 2017 [3]. Along with impairments in mood and cognition, and related decrements in work and social functioning, depressive disorders are associated with substantial healthcare resource use and economic burden [1, 2]. Estimates of direct health expenditures associated with adult depression exceed $90 billion in the US; further costs related to suicide and workplace problems were estimated to exceed $100 billion [2].

The Emergency Department (ED) is a frequently used care setting by patients with mental disorders [4]. Nationwide data from 1992 to 2000 showed increasing ED visits for psychiatric reasons in the US, indicating patients with psychiatric diagnoses had a higher admission rate than patients without such diagnoses [4]. This analysis [4] did not specify ED visits for patients with depression. More recent work by Ballou et al. analyzed 2 years (2006 and 2014) of national data on US ED visits for depressive symptoms, finding the rate of these visits to be greater in 2014 [5]. However, to date, data on adults with depression presenting to the ED for all reasons (i.e., not strictly for psychiatric emergencies) have received limited attention [6]. In comparison to other diseases such as cancer and diabetes, the populational characteristics of ED patients with depression and their needs for ED care and services are yet to be investigated [7, 8]. More targeted treatment of depressed patients in the ED through evidence-based interventions may be needed. We seek to inform efforts to improve the quality of ED care delivered to patients with depression and to reduce potentially unnecessary ED use.

By means of a secondary analysis of a large, nationally representative dataset, the current study aims to estimate ED utilization by patients with depression, describe the clinical presentation of patients with depression in the ED setting, and examine factors associated with clinical outcomes and resource utilization in this population.

## Methods

This is a cross-sectional study of ED data obtained from a multiyear, nationally representative survey carried out in the US. This study used preexisting, deidentified data and thus was categorized as exempt by the University of Michigan’s Institutional Review Board.

### Study population

The study population consisted of all adult patients (age ≥ 18 years) (*N* = 42,832; Weighted *N* = 278,699,057) in the National Hospital Ambulatory Medical Care Survey Emergency Department Subfile (NHAMCS-ED) from 2014 to 2016 [9]. NHAMCS-ED is a nationally representative, multistage, stratified probability sample of ED visits in the United States, administered by the National Center for Health Statistics, a branch of the Centers for Disease Control and Prevention. The NHAMCS-ED sample is collected from approximately 300 hospital-based EDs per year, randomly selected from approximately 1900 geographic areas in all 50 states. The survey uses a standardized data collection form to gather detailed information from approximately 100 patients per hospital-based ED.

### Study variables

The primary outcome used for the study was the patients’ depression status (whether the patients currently have depression identified by the variable “depression status” in the NHAMC-ED subfile). This status includes “affective disorders and major depressive disorders, such as episodes of depressive reaction, psychogenic depression, and reactive depression” (p. 148) [10].

Secondary outcomes included the Emergency Severity Index (ESI) score (a five-level ED triage algorithm assigning patients a score from 1 [most urgent] to 5 [least urgent] on the basis of acuity and resource needs),hospital admission, intensive care unit (ICU) admission,blood test, imaging (including X-ray, CT, ultrasound, and MRI), procedures (BiPAP/CPAP; bladder catheter; cast, splint, wrap; central line other; IV fluids; CPR; endotracheal intubation; incision & drainage; IV fluids; lumbar puncture; nebulizer therapy; pelvic exam; skin adhesives; suturing/staples; other),whether the patient left before triage or treatment, length of stay, and whether the patient died in the ED/hospital.

Covariates included demographic characteristics (age, sex, race/ethnicity, region); socioeconomic status indicators, including residence (private home, nursing home, homeless, other) and insurance (private insurance, Medicare, Medicaid/CHIP, uninsured, other); day and mode of arrival; triage vital signs (temperature, pain scale, blood pressure, etc.); and reason for the ED visit. To assign a primary reason for each ED visit, we synthesized ten system-based symptom clusters from the nine symptom modules used in the NHAMCS (for reference, see p. 164 [10]). The detail of the coding algorithms and categories were introduced in the Appendicitis II of each years’ survey file documentations. Generally, the reason for visit was coded into 8 main modules including symptom module, disease module, diagnostic module, screening and preventive module, treatment module, injuries and adverse effects module, test results module, and administrative module. In the current study, we used the symptom module, which were categorized into 10 groups including (1) general symptoms; (2) symptoms referable to psychological and mental disorders; (3) symptoms referable to the nervous system; (4) symptoms referable to the cardiovascular and lymphatic systems; (5) symptoms referable to the eyes and ears; (6) symptoms referable to the respiratory system; (7) symptoms referable to the digestive system; (8) symptoms referable to the genitourinary system; (9) symptoms referable to the skin, nails, and hair; (10) symptoms referable to the musculoskeletal system. Note that, as per the NHAMCS modules, our “Reason for ED Visit – Psychiatric” cluster excluded the following: alcoholism, adverse effects of alcohol, drug (prescription and illicit) addiction/dependence, drug intoxication, intentional drug overdose, and unintentional overdose.

### Statistical analyses

Population characteristics between ED patients with versus those without depression were described and compared using χ^2^ tests. We used logistic regression to test for associations between the depression status and the covariates after adjusting for confounding factors. We also used logistic regression to investigate associations between depression and secondary outcomes, testing for mediation by covariates. The NHAMCS-ED dataset used in this analysis relies on a sequential hot-deck method to impute 3-digit ICD-9-CM codes (ICD-10-CM for 2016) for items such as age, sex, primary diagnosis, ED volume, and geographic region. Other variables were imputed with the median of the corresponding variables prior to generating the logistic regression models. We used SAS® (Version 9.4) for our analysis, setting α = 0.05 as the statistical significance threshold. All odds ratios were calculated with 95% confidence intervals; *p*-values are < 0.05 unless otherwise stated.

## Results

In 2014–2016, there were 278,699,057 total adult ED visits in the US, corresponding to approximately 92,899,685 annual ED visits. Patients with depression made up approximately 31,878,551 (11.4%) (10,626,184 annually) of these visits. Basic characteristics are described in Table 1.

**Table 1:** Baseline Characteristics of Patients Presenting to the ED, Stratified by Depression, NHAMCS 2014–2016

	Unweighted Sample (%)			Weighted Sample (%)		
	All	No Depression	Depression	All	No Depression	Depression
	42,832	37,737 (88.1)	5059 (11.9)	278,699,057	246,820,506 (88.6)	31,878,551 (11.4)
Male*	18,469 (43.1)	16,649 (44.1)	1820 (35.7)	119,751,766 (43.0)	109,011,905 (44.2)	10,739,861 (33.7)
Age*						
18–39	17,912 (41.8)	16,085 (42.6)	1827 (35.9)	118,068,691 (42.4)	106,669,971 (43.2)	11,398,720 (35.8)
40–49	6662 (15.6)	5727 (15.2)	935 (18.4)	43,185,040 (15.5)	37,429,025 (15.2)	5,756,015 (18.1)
50–59	6707 (15.7)	5704 (15.1)	1003 (19.7)	42,679,091 (15.3)	36,593,175 (14.8)	6,085,916 (19.1)
60–74	6678 (15.6)	5807 (15.4)	871 (17.1)	43,420,164 (15.6)	37,739,318 (15.3)	5,680,845 (17.8)
≥ 75	4873 (11.4)	4414 (11.7)	459 (9.0)	31,346,071 (11.2)	28,389,016 (11.5)	2,957,055 (9.3)
Race/ethnicity*						
White	27,251 (63.6)	23,546 (62.4)	3705 (72.7)	175,775,546 (63.1)	152,171,847 (61.7)	23,603,698 (74.0)
Black	9207 (21.5)	8299 (22.0)	908 (17.8)	62,663,628 (22.5)	57,104,372 (23.1)	5,559,256 (17.4)
Hispanic	5152 (12.0)	4754 (12.6)	398 (7.8)	33,391,671 (12.0)	31,036,082 (12.6)	2,355,589 (7.4)
Asian	804 (1.9)	760 (2.0)	44 (0.9)	4,392,213 (1.6)	4,221,497 (1.7)	170,717 (0.5)
Other	418 (1.0)	378 (1.0)	40 (0.8)	2,475,999 (0.9)	2,286,708 (0.9)	189,291 (0.6)
Residence type*						
Private residence	39,819 (95.1)	35,290 (95.6)	4529 (91.3)	258,354,513 (95.3)	230,189,300 (95.7)	28,165,213 (91.7)
Nursing home	885 (2.1)	703 (1.9)	182 (3.7)	5,875,161 (2.2)	4,597,878 (1.9)	1,277,283 (4.2)
Homeless	534 (1.3)	392 (1.1)	142 (2.9)	2,480,109 (0.9)	1,835,949 (0.8)	644,160 (2.1)
Other	651 (1.6)	541 (1.5)	110 (2.2)	4,501,686 (1.7)	3,885,684 (1.6)	616,002 (2.0)
Insurance type*						
Private insurance	12,446 (30.8)	11,293 (31.7)	1153 (23.9)	79,443,111 (30.5)	72,336,078 (31.3)	7,107,033 (24.1)
Medicare	10,517 (26.0)	8972 (25.2)	1545 (32.1)	66,956,323 (25.7)	57,073,540 (24.7)	9,882,783 (33.5)
Medicaid or CHIP	11,148 (27.6)	9467 (26.6)	1681 (34.9)	71,529,605 (27.5)	61,857,136 (26.8)	9,672,469 (32.8)
Uninsured	4886 (12.1)	4564 (12.8)	322 (6.7)	33,248,283 (12.8)	31,145,408 (13.5)	2,102,876 (7.1)
Other	1406 (3.5)	1289 (3.6)	117 (2.4)	9,371,908 (3.6)	8,660,813 (3.7)	711,095 (2.4)
Year*						
2014	15,319 (35.8)	13,677 (36.2)	1642 (32.2)	90,554,699 (32.5)	81,304,754 (32.9)	9,249,945 (29.0)
2015	14,041 (32.8)	12,364 (32.8)	1677 (32.9)	89,005,064 (31.9)	78,981,947 (32.0)	10,023,117 (31.4)
2016	13,472 (31.5)	11,696 (31.0)	1776 (34.9)	99,139,294 (35.6)	86,533,805 (35.1)	12,605,489 (39.5)
Day of ED Visit						
Sunday	5622 (13.1)	4964 (13.2)	658 (12.9)	35,918,011 (12.9)	31,810,077 (12.9)	4,107,933 (12.9)
Monday	6930 (16.2)	6146 (16.3)	784 (15.4)	44,958,717 (16.1)	39,878,806 (16.2)	5,079,911 (15.9)
Tuesday	6347 (14.8)	5614 (14.9)	733 (14.4)	40,922,676 (14.7)	36,423,291 (14.8)	4,499,385 (14.1)
Wednesday	6225 (14.5)	5489 (14.5)	736 (14.4)	40,888,226 (14.7)	36,088,222 (14.6)	4,800,003 (15.1)
Thursday	5952 (13.9)	5211 (13.8)	741 (14.5)	39,069,043 (14.0)	34,301,499 (13.9)	4,767,544 (15.0)
Friday	5960 (13.9)	5253 (13.9)	707 (13.9)	38,869,467 (13.9)	34,729,896 (14.1)	4,139,571 (13.0)
Saturday	5796 (13.5)	5060 (13.4)	736 (14.4)	38,072,918 (13.7)	33,588,715 (13.6)	4,484,203 (14.1)
Arrive by ambulance*	7729 (18.5)	6462 (17.6)	1267 (25.4)	49,769,047 (18.3)	41,864,145 (17.4)	7,904,902 (25.3)
Seen within last 72 h	1914 (4.9)	1668 (4.9)	246 (5.3)	11,953,039 (4.8)	10,393,280 (4.7)	1,559,759 (5.5)
Pain level at Presentation						
No pain	7711 (24.4)	6654 (23.8)	1057 (28.5)	46,478,004 (23.1)	40,809,768 (22.9)	5,668,235 (25.2)
Mild	2916 (9.2)	2667 (9.5)	249 (6.7)	18,235,636 (9.1)	16,637,092 (9.3)	1,598,544 (7.1)
Moderate	9430 (29.8)	8429 (30.2)	1001 (27.0)	60,509,861 (30.1)	54,315,526 (30.4)	6,194,335 (27.6)
Severe	11,602 (36.6)	10,203 (36.5)	1399 (37.7)	75,762,102 (37.7)	66,762,362 (37.4)	8,999,740 (40.1)
Temperature at Presentation						
> 36 °C–38 °C	38,083 (94.6)	33,546 (94.7)	4537 (94.4)	249,171,894 (95.1)	220,726,403 (95.1)	28,445,491 (94.9)
≤ 36 °C	1522 (3.8)	1307 (3.7)	215 (4.5)	9,089,224 (3.5)	7,856,127 (3.4)	1,233,097 (4.1)
> 38 °C	635 (1.6)	581 (1.6)	54 (1.1)	3,863,922 (1.5)	3,583,001 (1.5)	280,921 (0.9)
Heart Rate at Presentation						
≤ 90	28,489 (66.5)	25,321 (67.1)	3168 (62.2)	184,822,552 (66.3)	164,924,803 (66.8)	19,897,749 (62.4)
90–100	7169 (16.7)	6203 (16.4)	966 (19.0)	46,314,663 (16.6)	40,458,700 (16.4)	5,855,963 (18.4)
100–110	3906 (9.1)	3394 (9.0)	512 (10.0)	25,427,295 (9.1)	22,226,080 (9.0)	3,201,215 (10.0)
110–120	1988 (4.6)	1723 (4.6)	265 (5.2)	13,118,183 (4.7)	11,441,804 (4.6)	1,676,379 (5.3)
> 120	1280 (3.0)	1096 (2.9)	184 (3.6)	9,016,363 (3.2)	7,769,119 (3.1)	1,247,244 (3.9)
DBP at Presentation						
60–80	19,358 (45.2)	17,041 (45.2)	2317 (45.5)	125,677,278 (45.1)	111,249,212 (45.1)	14,428,067 (45.3)
< 60	4312 (10.1)	3791 (10.0)	521 (10.2)	26,198,088 (9.4)	23,124,144 (9.4)	3,073,944 (9.6)
> 80	19,162 (44.7)	16,905 (44.8)	2257 (44.3)	126,823,690 (45.5)	112,447,150 (45.6)	14,376,540 (45.1)
SBP at Presentation						
80–120	9773 (22.8)	8509 (22.5)	1264 (24.8)	61,351,488 (22.0)	53,148,943 (21.5)	8,202,545 (25.7)
< 80	1588 (3.7)	1412 (3.7)	176 (3.5)	9,419,022 (3.4)	8,412,329 (3.4)	1,006,693 (3.2)
> 120	31,471 (73.5)	27,816 (73.7)	3655 (71.7)	207,928,547 (74.6)	185,259,235 (75.1)	22,669,312 (71.1)
Census Region**						
Northeast	7176 (16.8)	6200 (16.4)	976 (19.2)	43,967,048 (15.8)	38,060,463 (15.4)	5,906,584 (18.5)
Midwest	10,893 (25.4)	9337 (24.7)	1556 (30.5)	74,304,118 (26.7)	63,492,646 (25.7)	10,811,472 (33.9)
South	15,430 (36.0)	13,862 (36.7)	1568 (30.8)	105,760,507 (37.9)	95,725,338 (38.8)	10,035,169 (31.5)
West	9333 (21.8)	8338 (22.1)	995 (19.5)	54,667,385 (19.6)	49,542,059 (20.1)	5,125,326 (16.1)
ED Visit Classification**						
Injury/trauma	12,286 (30.1)	10,964 (30.5)	1322 (27.3)	78,178,483 (29.5)	70,073,749 (29.8)	8,104,734 (26.7)
Overdose/poisoning	499 (1.2)	364 (1.0)	135 (2.8)	3,358,380 (1.3)	2,574,059 (1.1)	784,322 (2.6)
Adverse effect of medical/surgical treatment	1099 (2.7)	949 (2.6)	150 (3.1)	7,170,683 (2.7)	6,202,919 (2.6)	967,764 (3.2)
Visit not related to any above	26,692 (65.4)	23,506 (65.4)	3186 (65.9)	174,903,611 (66.0)	154,777,524 (65.9)	20,126,087 (66.4)
Unknown Injury Status	214 (0.5)	170 (0.5)	44 (0.9)	1,546,669 (0.6)	1,219,470 (0.5)	327,199 (1.1)

*Note*: the missing proportion for arrival mode, residency type, and injury is less than 5%; for insurance, temperature, heart rate, and blood pressure, and seen within last 72 h, 5–10%; for triage level, 27%; for pain level, 26%

*p < 0.05, **p < 0.01

The proportion of ED visits by patients with depression varied by US census region: Northeast, 18.5%; Midwest, 33.9%; South, 31.5%; and West, 16.1% (*p* < 0.01). A greater proportion of ED patients with depression belonged to the 40–49, 50–59, and 60–74 age groups as compared to their non-depressed counterparts (18.1 vs. 15.2%, 19.1 vs. 14.8%, and 17.8 vs. 15.3%, respectively). ED patients with depression comprised a higher proportion of non-Hispanic Whites relative to those without depression (74.0 vs. 61.7%).

Table 2 describes associations between ED patients’ characteristics (demographic, socioeconomic, and clinical) and their depression status. Male ED patients were 38% (aOR: 0.62; CI: 0.59–0.67) less likely than females to have depression. Among ED patients, Blacks were 38% (aOR: 0.62; CI: 0.57–0.68) less likely than Whites to have depression; Hispanics, 49% less likely (aOR:0.51; CI: 0.45–0.57); and Asians, 63% less likely (aOR:0.37 CI: 0.27–0.51). Compared to ED patients inhabiting a private residence, those who were living in nursing homes or were homeless were 1.63(95% CI: 1.35–1.97) and 1.82 (CI: 1.46–2.26) times, respectively, more likely to have depression. Compared to ED patients with private insurance, those with Medicare and Medicaid or CHIP were 1.82 (95% CI: 1.66–2.00) and 1.74 times (CI: 1.60–1.89), respectively, more likely to have depression. Compared to patients who arrived at the ED by other means, patients who arrived by ambulance were 1.25 times more likely (95% CI: 1.16–1.36) to have depression. ED patients who presented with overdose/poisoning were 2.19 times (CI: 1.75–2.74) more likely to have depression than those presenting with injury or trauma. ED patients who sought care for psychiatric reasons were 5.00 times (95% CI: 4.41–5.66) more likely to have depression than those seeking care for general symptoms (Table 3). Among ED patients with depression, males were 2.45 (95% CI: 2.10–2.87) times more likely than females to be seeking emergency care for psychiatric reasons and were 1.46 (95% CI 1.03–2.05) times more likely than females to present with overdose/poisoning.

**Table 2:** Association Between Depression Status in ED Patients and Their Visiting Characteristics (NHAMCS 2014–2016)

	Crude OR (95% CI)	Adjusted OR (95% CI)
Age		
18–39	Reference [1]	Reference [1]
40–49	1.44(1.32–1.56)	1.41(1.29–1.55)
50–59	1.55(1.43–1.68)	1.48(1.35–1.62)
60–74	1.32(1.21–1.44)	1.03(0.93–1.14)
≥ 75	0.92(0.82–1.02)	0.52(0.45–0.60)
Male vs. Female	0.70(0.66–0.75)	0.62(0.59–0.67)
Race/ethnicity		
White	Reference [1]	Reference [1]
Black	0.70(0.64–0.75)	0.62(0.57–0.68)
Hispanic	0.53(0.48–0.59)	0.51(0.45–0.57)
Asian	0.37(0.27–0.50)	0.37(0.27–0.51)
Other	0.67(0.49–0.93)	0.65(0.46–0.91)
Day of Week		
Sunday	Reference [1]	Reference [1]
Monday	0.96(0.86–1.07)	0.95(0.85–1.07)
Tuesday	0.99(0.88–1.10)	0.94(0.84–1.06)
Wednesday	1.01(0.90–1.13)	0.99(0.88–1.12)
Thursday	1.07(0.96–1.20)	1.06(0.94–1.19)
Friday	1.02(0.91–1.14)	0.97(0.86–1.09)
Saturday	1.10(0.98–1.23)	1.07(0.95–1.21)
Year		
2014	Reference [1]	Reference [1]
2015	1.13(1.05–1.21)	1.09(1.01–1.18)
2016	1.27(1.18–1.36)	1.22(1.13–1.32)
Residence type		
Private residence	Reference [1]	Reference [1]
Nursing home	2.01(1.70–2.37)	1.63(1.35–1.97)
Homeless	2.81(2.31–3.41)	1.82(1.46–2.26)
Other	1.58(1.28–1.94)	1.29(1.03–1.61)
Insurance type		
Private insurance	Reference [1]	Reference [1]
Medicare	1.60(1.48–1.74)	1.82(1.66–2.00)
Medicaid or CHIP	1.74(1.61–1.88)	1.74(1.60–1.89)
Uninsured	0.69(0.61–0.79)	0.76(0.66–0.87)
Other	0.89(0.73–1.08)	1.03(0.84–1.26)
Temperature		
36 °C–38 °C	Reference [1]	Reference [1]
≤ 36 °C	1.22(1.06–1.42)	1.17(1.00–1.36)
> 38 °C	0.69(0.52–0.92)	0.65(0.49–0.87)
Heart Rate		
≤ 90	Reference [1]	Reference [1]
90–100	1.25(1.15–1.35)	1.20(1.10–1.30)
100–110	1.21(1.09–1.33)	1.11(1.00–1.24)
110–120	1.23(1.08–1.41)	1.13(0.98–1.30)
> 120	1.34(1.14–1.58)	1.21(1.02–1.44)
DBP		
60–80	Reference [1]	Reference [1]
< 60	1.01(0.91–1.12)	0.98(0.88–1.09)
> 80	0.98(0.92–1.05)	0.94(0.88–1.01)
Pain level		
No pain	Reference [1]	Reference [1]
Mild	0.59(0.51–0.68)	0.77(0.66–0.90)
Moderate	0.83(0.76–0.89)	0.98(0.90–1.07)
Severe	0.86(0.79–0.94)	1.10(1.00–1.22)
72-h revisit vs. not	0.91(0.80–1.05)	0.90(0.78–1.04)
Arrival by Ambulance vs. Not	1.60(1.50–1.72)	1.25(1.16–1.36)
Census Region		
Northeast	Reference [1]	Reference [1]
Midwest	1.06(0.97–1.15)	1.08(0.99–1.19)
South	0.72(0.66–0.78)	0.81(0.74–0.89)
West	0.76(0.69–0.83)	0.79(0.72–0.88)
Reason for Visit (by Symptom Module)		
General	Reference [1]	Reference [1]
Psychiatric	5.03(4.48–5.65)	5.00(4.41–5.66)
Neurologic	0.87(0.76–0.98)	0.82(0.72–0.93)
Cardiovascular and Lymphatic	0.66(0.52–0.83)	0.68(0.54–0.87)
Eyes and/or Ears	0.55(0.42–0.71)	0.59(0.45–0.77)
Respiratory	0.78(0.69–0.88)	0.77(0.68–0.87)
Digestive	0.79(0.71–0.87)	0.78(0.70–0.87)
Genitourinary	0.58(0.49–0.68)	0.60(0.50–0.70)
Dermatologic	0.56(0.45–0.69)	0.60(0.48–0.75)
Musculoskeletal	0.72(0.65–0.80)	0.75(0.67–0.84)
Other	0.82(0.74–0.90)	0.88(0.78–0.99)
Reason for Visit (by Injury Type)		
Injury/Trauma	Reference [1]	Reference [1]
Overdose/Poisoning	3.08(2.51–3.78)	2.19(1.75–2.74)
Adverse effect of medical/surgical treatment	1.31(1.09–1.57)	1.23(1.01–1.48)
Visit not related to any above	1.13(1.06–1.21)	1.12(1.03–1.22)
Questionable injury status	2.15(1.53–3.01)	1.45(1.01–2.09)

*Note*: the adjusted OR was from a logistic regression including all variables in the table

**Table 3:** Selected Reason for Visit and Emergency Department Diagnosis among ED Patients with Depression, NHAMCS 2014–2016

Reason for ED Visit	Unweighted Sample (%)			Weighted Sample (%)		
	All	No Depression	Depression	All	No Depression	Depression
General	8187 (19.1)	7126 (18.9)	1061 (20.8)	53,664,580 (19.3)	46,862,329 (19.0)	6,802,251 (21.4)
Psychiatric	1700 (4.0)	972 (2.6)	728 (14.3)	9,426,523 (3.4)	5,550,218 (2.3)	3,876,305 (12.2)
Neurologic	3304 (7.7)	2927 (7.8)	377 (7.4)	20,833,741 (7.5)	18,542,430 (7.5)	2,291,311 (7.2)
Cardiovascular and Lymphatic	889 (2.1)	810 (2.2)	79 (1.6)	5,993,917 (2.2)	5,512,635 (2.2)	481,282 (1.5)
Eyes and/or Ears	848 (2.0)	784 (2.1)	64 (1.3)	5,778,778 (2.1)	5,233,633 (2.1)	545,145 (1.7)
Respiratory	4198 (9.8)	3762 (10.0)	436 (8.6)	27,856,021 (10.0)	25,021,474 (10.2)	2,834,547 (8.9)
Digestive	6807 (15.9)	6093 (16.2)	714 (14.0)	46,038,272 (16.5)	41,187,597 (16.7)	4,850,675 (15.2)
Genitourinary	2477 (5.8)	2281 (6.1)	196 (3.9)	14,984,361 (5.4)	13,934,779 (5.7)	1,049,581 (3.3)
Dermatologic	1333 (3.1)	1231 (3.3)	102 (2.0)	8,716,118 (3.1)	8,018,952 (3.3)	697,166 (2.2)
Musculoskeletal	6519 (15.2)	5891 (15.6)	628 (12.3)	42,820,579 (15.4)	38,855,925 (15.8)	3,964,654 (12.5)
Other	6501 (15.2)	5796 (15.4)	705 (13.9)	42,147,135 (15.1)	37,695,947 (15.3)	4,451,188 (14.0)

Tables 4 and 5 describe the proportions of ESI, hospital admission, ICU admission, and medical resources utilization, stratified by depression. In the weighted sample, 17.8% of the patients with depression were assigned immediate or emergent ESI score, which was higher than the patients with no depression (11.9%). A total of 21.1% of the patients with depression were admitted into hospital after the ED visit, which was higher than the patients with no depression (12%). A total of 2.6% of patients with depression were admitted into ICU, while the proportion for the patients with no depression was 1.6%. Moreover, 59.8% of the patients with depression obtained a blood test, which is higher than the proportion for patients without depression (50.1%). The hospital admission rate among ED patients was 1.50 times higher for patients with depression (CI: 1.38–1.63); depressed patients were also 1.33 times more likely to receive immediate vs. semi- or non-urgent ESI scores (CI: 1.18–1.49) compared to patients without depression. ED patients with depression were 1.33 (CI: 1.24–1.43) times more likely to receive blood tests but were less likely to access other resources medical resources; for example, they were 18% (CI: 0.59–1.14) less likely to receive an MRI scan as compared to patients without depression.

**Table 4:** Proportion of Emergency Severity Index, Hospital admission, ICU admission, Medical resources utilization, stratified by Depression, NHAMCS 2014–2016

	Unweighted Sample			Weighted Sample		
	All	No Depression	Depression	All	No Depression	Depression
ESI score**						
1 (Immediate)	239 (0.8)	210 (0.8)	29 (0.8)	1,496,327 (0.8)	1,272,228 (0.7)	224,099 (1.0)
2 (Emergent)	3615 (11.6)	2966 (10.9)	649 (16.9)	23,433,327 (11.8)	19,516,968 (11.2)	3,916,359 (16.8)
3 (Urgent)	15,392 (49.5)	13,428 (49.3)	1964 (51.0)	97,000,149 (49.0)	85,337,086 (48.8)	11,663,063 (50.1)
4 (Semi-urgent)	10,051 (32.3)	9037 (33.2)	1014 (26.3)	65,085,335 (32.9)	58,899,226 (33.7)	6,186,109 (26.6)
5 (Non-urgent)	1784 (5.7)	1589 (5.8)	195 (5.1)	11,046,598 (5.6)	9,768,544 (5.6)	1,278,054 (5.5)
Hospital Admission	5852 (13.7)	4764 (12.6)	1088 (21.4)	36,388,538 (13.1)	29,653,166 (12.0)	6,735,373 (21.1)
ICU*	698 (1.6)	587 (1.6)	111 (2.2)	4,647,353 (1.7)	3,833,519 (1.6)	813,835 (2.6)
Death in ED or hospital	201 (0.5)	187 (0.5)	14 (0.3)	1,342,510 (0.5)	1,224,939 (0.5)	117,571 (0.4)
Left before/after triage*	1085 (2.5)	978 (2.6)	107 (2.1)	6,792,175 (2.4)	6,134,329 (2.5)	657,846 (2.1)
Blood test performed*	21,958 (51.3)	18,860 (50.0)	3098 (60.8)	142,656,097 (51.2)	123,598,652 (50.1)	19,057,445 (59.8)
Any imaging performed	21,950 (51.2)	19,494 (51.7)	2456 (48.2)	144,824,612 (52.0)	128,979,436 (52.3)	15,845,177 (49.7)
X-ray in ED	15,099 (35.3)	13,363 (35.4)	1736 (34.1)	99,429,274 (35.7)	88,271,718 (35.8)	11,157,556 (35.0)
CT in ED	8414 (19.6)	7392 (19.6)	1022 (20.1)	54,986,804 (19.7)	48,528,197 (19.7)	6,458,608 (20.3)
Ultrasound in ED	2218 (5.2)	2012 (5.3)	206 (4.0)	14,936,538 (5.4)	13,580,831 (5.5)	1,355,707 (4.3)
MRI in ED*	446 (1.0)	402 (1.1)	44 (0.9)	2,831,626 (1.0)	2,594,364 (1.1)	237,262 (0.7)
Other Imaging in ED	604 (1.4)	539 (1.4)	65 (1.3)	4,297,097 (1.5)	3,896,319 (1.6)	400,778 (1.3)
Procedure	21,021 (49.1)	18,626 (49.4)	2395 (47.0)	133,801,012 (48.0)	119,211,149 (48.3)	14,589,863 (45.8)
Waiting time (min, means [95% CI])	41.1 (40.3–41.8)	41.0 (40.2–41.8)	41.7 (39.3–44.1)	39.9 (39.2–40.6)	39.8 (39.0–40.5)	41.0 (38.9–43.2)
Visit length** (min, means [95% CI])	245.6 (241.6–249.6)	237.5 (233.4–241.6)	309.1 (293.5–324.6)	230.2 (226.7–233.8)	224.8 (221.2–228.5)	275.2 (262.4–288.0)

*Note*: “Waiting time” is time from arrival to seeing the physician; “length of visit” is time from arrival to discharge. *p < 0.05; **p < 0.01

**Table 5:** Odds Ratio of Emergency Severity Index, Hospital Admission, ICU Admission, Medical Resources Utilization for Patients with versus without Depression, NHAMCS 2014–2016

	Crude Odds Ratio	*Adjusted for variables*^a^		
		Demographic	+ Socioeconomic	+ Visiting & Clinical
ESI Score: 1 or 2 vs. 4 or 5	1.88(1.69–2.08)	1.93(1.74–2.14)	1.85(1.67–2.06)	1.33(1.18–1.49)
ESI Score: Urgent vs. Semi- or Non-Urgent	1.29(1.19–1.39)	1.28(1.18–1.38)	1.25(1.16–1.35)	1.11(1.02–1.20)
Hospital Admission	1.88(1.75–2.02)	2.01(1.86–2.17)	1.84(1.70–1.99)	1.50(1.38–1.63)
ICU	1.41(1.15–1.73)	1.46(1.19–1.80)	1.32(1.07–1.63)	1.10(0.88–1.37)
Death	0.55(0.32–0.95)	0.62(0.36–1.07)	0.58(0.33–1.00)	0.43(0.24–0.77)
Left	0.81(0.66–0.99)	0.81(0.66–0.99)	0.80(0.65–0.98)	0.77(0.62–0.95)
Blood test	1.55(1.46–1.65)	1.54(1.45–1.63)	1.52(1.43–1.61)	1.33(1.24–1.43)
Any imaging	0.87(0.82–0.92)	0.85(0.80–0.90)	0.84(0.79–0.90)	0.89(0.84–0.95)
X-ray	0.94(0.89–1.00)	0.93(0.87–0.99)	0.92(0.87–0.99)	0.95(0.89–1.02)
CT	1.03(0.96–1.11)	1.02(0.95–1.10)	1.01(0.94–1.09)	0.97(0.89–1.05)
Ultrasound	0.75(0.65–0.87)	0.73(0.63–0.84)	0.74(0.64–0.86)	0.84(0.71–0.98)
MRI	0.81(0.59–1.11)	0.79(0.58–1.08)	0.84(0.61–1.16)	0.82(0.59–1.14)
Procedure	0.90(0.85–0.96)	0.90(0.85–0.96)	0.90(0.85–0.96)	0.92(0.86–0.97)

^a^“Demographic” includes gender, age group, and race/ethnicity; “socioeconomic” includes residence type, insurance type, and census region; “visiting & clinical” includes year, day of the week, arrival by ambulance, seen within last 72 h, pain level, temperature, heart rate, dialytic blood pressure, injury status, and reason for visit

## Discussion

To our knowledge, this is the first, most comprehensive study describing the national characteristics of ED patients with depression. An insightful study by Ballou et al. [5] looked at US ED visits by depressed patients, but there are key differences between their work and ours. Namely, Ballou et al.’s scope was limited to ED visits for depressive complaints; further, they analyzed ED data from a different database (the Nationwide Emergency Department Sample), sampling 2 years (2006 and 2014) of data [5]. Studies on general ED use by patients with depression have been more limited than the present study in terms of sample size and national representativeness. For example, a prospective cohort study by Beiser et al. included a comparatively small sample (*n* = 999), as did Kumar et al.’s study of depression prevalence assessed in ED admissions (*n* = 536) [11, 12]. Beiser et al. and Kumar et al. reported markedly higher rates of depression (27 and 30%, respectively) among ED patients than the rate noted here (11.4%) [11, 12]. However, we must point out methodologic differences between their studies [11, 12] and ours. Whereas we used NHAMCS data on ED patients’ depression status, these studies [11, 12] relied on self-reported depression questionnaires administered to patients in their ED. Further, our study characterizes ED patients with depression with greater power by using 3 years of data from a larger, more representative sample. Our study also provides previously unreported data concerning vitals and other clinical information in ED patients with depression.

From 2014 to 2016, patients with depression made more than 10 million ED visits annually. Compared to patients without depression, those with depression had higher rates of hospital admission and ICU admission. We did not observe associations between ED patients’ depression and any somatic reasons for ED visit. This finding suggests that comorbid conditions that have previously been correlated with depression (e.g., rheumatoid arthritis) may not strongly predict these patients’ emergency care needs [12]. However, patients with depression were more likely to seek emergency treatment for psychiatric symptoms and for acute overdose/poisoning.

Demographic factors were associated with the prevalence of depression in ED patients. In terms of region, EDs in the Midwest had the greatest proportion of visits by patients with depression. Female ED patients were more likely than males to have depression, as were non-Hispanic Whites compared to other races/ethnicities, particularly Asians. These gender and racial/ethnic differences are roughly concordant with US demographic patterns in depression prevalence observed beyond the ED setting [13]. However, these patterns have been problematized by research indicating that certain non-White minority populations are less likely to receive or seek mental health diagnoses and care [14–16]. If the large proportion of Whites among patients with depression partly reflects such differences in diagnosis and treatment seeking, there may in fact be a number of non-White patients with unaddressed mental health care needs in the ED.

Notably, patients with depression who sought ED treatment for psychiatric symptoms were roughly two times more likely to be male than female, consistent with findings by Ballou et al. [5]. Because our psychiatric ED visit classification excluded visits related to alcoholism and other substance use disorders, our finding of higher rates of psychiatric ED visits for depressed male patients cannot be well explained by males’ higher rates of alcoholism and substance use disorders in the general population [3]. Given the social barriers that men, relative to women, face in seeking mental health care, this disparity may indicate a need for more routine depression care and/or screening in men to reduce their rate of psychiatric emergencies [17, 18]. Further, in Ballou et al.’s study, men were modestly more likely to present to the ED with self-harm [5]. In our sample, males with depression were more likely to present with overdose/poisoning—a finding that does not necessarily point to increased self-harm but bears highlighting for future inquiry. Additional data are needed to clarify the reasons for and extent of these gender-based disparities.

Compared to their non-depressed counterparts, patients with depression in the ED have higher ESI scores, hospital admission, and ICU admission, indicating that patients with depression require a higher level of emergency care. With regard to these outcomes, it is worth noting similarities between ED patients with depression and ED patients with cancer, whose utilization is higher across many dimensions of care [7, 19]. ED patients with cancer were also had higher odds of having depression [20]. The factors contributing to these outcomes in both patient populations may be the subject of future research. Understanding the reasons for ED revisits among patients with depression may facilitate the development of interventions or guidelines to reduce ED visit and revisit rates. Considering the substantial number of patients with depression in the ED, we suggest that the ED is an understudied setting for depression treatment. Finer-grained data on patients with a history of depression may inform ways of increasing this population’s use of routine care over emergency care options.

### Limitations

In the patient histories documented in the NHAMCS-ED data, patients are coded as either having or not having “depression status.” Information including depression severity, subtype, and duration were not specified in this dataset. Such information may have predictive value; for example, in Beiser et al.’s prospective cohort study, a 10% increase in depression severity was correlated with a 10% increase in future ED visits [11]. Further, apart from depression, the NHAMCS-ED dataset does not indicate other mental disorders in the ED patient sample. We therefore could not adjust for potential psychiatric comorbidities. More generally, other comorbidities that are not documented in the dataset, such as chronic pain disorders, could play a role in mediating some of the associations we observed [21]. Another limitation of the NHAMCS-ED dataset is a lack of information on treatment history (pharmacologic or otherwise) for depression. Future studies may examine ED patients’ specific depression characteristics, comorbidities, treatment history, and other finer-grained clinical data allowing for more refined associative and predictive models.

## Conclusions

Our study advances understanding of the characteristics and clinical presentation of patients with depression in the ED. It is an initial step toward improving their care and clinical outcomes and reducing this population’s ED burden. The study revealed the characteristics of ED patients with depression in a diverse, national sample. Patients with depression make more than 10 million ED visits annually. We found significant gender, age, racial/ethnic, and regional differences among these patients. In the ED, patients with depression have significantly higher hospital and ICU admission rates compared to those without depression, indicating that patients with depression may require a higher level of emergency care. These findings argue for increasing recognition of the potential of the ED as a high-leverage setting for improving treatment and screening of depression, by identifying characteristics and trajectories of patients presenting to the ED with depression.

## Data Availability

The NHAMCS-ED dataset can be accessed through the website of the US Centers for Disease Control and Prevention (CDC) (https://www.cdc.gov/nchs/ahcd/index.htm). The detailed explanation of the survey data for each year and the code book can be found here: https://ftp.cdc.gov/pub/Health_ Statistics/NCHS/dataset_documentation/nhamcs/.

## Abbreviations

aOR: Adjusted Odds Ratio
CI: Confidential Interval
ED: Emergency Department
NHAMCS-ED: National Hospital Ambulatory Medical Care Survey ED Subfile
ESI: Emergency Severity Index
ICU: Intensive care unit
CT: Computed tomography
MRI: Magnetic Resonance Imaging
